# Incidence rates of sickness absence related to mental disorders: a systematic literature review

**DOI:** 10.1186/1471-2458-14-205

**Published:** 2014-02-26

**Authors:** Carolyn S Dewa, Desmond Loong, Sarah Bonato, Hiske Hees

**Affiliations:** 1 Centre for Research on Employment and Workplace Health, Centre for Addiction and Mental Health, 33 Russell Street, Toronto M5S 2S1, Canada; 2 Library Services, Centre for Addiction and Mental Health, 33 Russell Street, Toronto M5S 2S1, Canada; 3 Department of Psychiatry, University of Toronto, 250 College Street, Toronto M5T 1R8, Canada; 4 Department of Mood Disorders, Pro Persona, Wagnerlaan 2, 6815 AG Arnhem, The Netherlands; 5 Department of Psychiatry, Academic Medical Center, University of Amsterdam, Meibergdreef 5, Room PA1-156, 1105 AZ Amsterdam, The Netherlands

**Keywords:** Sickness absence, Mental disorders, Incidence

## Abstract

**Background:**

Over the past decade, growing attention has been given to the mental health of workers. One way to examine the mental health of workers is to look at the incidence rates of mental illness-related sickness absence. There is a scarcity of literature in which the incidence rates of mental illness-related sickness absence among different countries have been considered together. The purpose of this systematic literature review is to address the question: Are there similarities and differences in the incidence rates of mental disorder-related sickness absence among and within OECD identified Social Democratic, Liberal and Latin American country categories? In this paper, we seek to identify differences and similarities in the literature rather than to explain them. With this review, we lay the groundwork for and point to areas for future research as well as to raise questions regarding reasons for the differences and similarities.

**Methods:**

A systematic literature search of the following databases were performed: *Medline Current*, *Medline In-process*, *PsycINFO*, *Econlit* and *Web of Science*. The search period covered 2002–2013. The systematic literature search focused on working adults between 18–65 years old who had not retired and who had mental and/or substance abuse disorders. Intervention studies were excluded. The search focused on medically certified sickness absences.

**Results:**

A total of 3,818 unique citations were identified. Of these, 10 studies met the inclusion/exclusion criteria; six were from Social Democratic countries. Their quality ranged from *good* to *excellent*. There was variation in the incidence rates reported by the studies from the Social Democratic, Liberal and Latin American countries in this review.

**Conclusions:**

The results of this systematic review suggest that this is an emerging area of inquiry that needs to continue to grow. Priority areas to support growth include cross jurisdictional collaboration and development of a typology characterizing the benefit generosity and work integration policies of sickness absence schemes. Finally, the literature should be updated to reflect changes in sickness absence benefit schemes over time.

## Background

### Global focus on worker mental health

Over the past decade, increasing attention has turned to the mental health of workers and its effects on the workplace. For example, European Ministers of Health have advocated that employers include mental health programs as part of occupational health and safety [1]. Similar endorsements have been made in Australia [2], the United States [3] and Canada [4].

The impetus for greater attention to worker mental health has also been spurred by a growing awareness of the impact of mental disorders on the workplace. Indeed, an expanding body of literature indicates that mental illness takes its workplace toll in the form of work absences and decreased productivity (e.g., [5-7]). Because of the length of their absences [8-12] and their rates of recurrence [10,13-15], sickness absences related to mental illness are one of the most costly types of sickness absences. Thus, there is an interest in promoting worker mental health.

### Importance of incidence rates of mental illness-related sickness absence

One way to examine the mental health of workers is to look at the incidence rates of mental illness-related sickness absence. That is, the better the mental health status of workers, the lower the incidence rates of mental illness-related sickness absence. But, because there are differences in the way various countries approach the mental health of their working populations [16], it could be useful to consider incidence rates by jurisdictions. Jurisdictions with higher rates might be places where further exploration could take place to identify the types of approaches to avoid. In contrast, those with lower rates may be places where further studies could be conducted to learn about effective practices.

### Considering country variations

One of the challenges of examining the rates of sickness absence incidence among countries is related to the heterogeneity of country system factors that affect workers. Examples of these system factors include country work integration policies such as employer sickness absence obligations, employment rehabilitation programs and work incentives. Another group of factors is related to country compensation policies such as the population covered, disability benefit eligibility and criteria.

Recognizing the heterogeneity among countries, the Organization for Economic Co-operation and Development (OECD) developed a classification system to be used to understand the similarity among countries with respect to their work integration and worker compensation policies. The classification builds on work from the political economy literature that was developed to compare social policies across diverse jurisdictions (e.g., [17-19]). The classification focuses on the types of public policies (e.g., work integration schemes) that would affect work-related outcomes (e.g., employment rates) [20]. The classification system facilitates discussion without becoming entrenched in the complexities of individual systems [19].

The OECD [16] examined the disability policies of 15 OECD countries. Disability policies were evaluated based on the generosity of their compensation and the extent of their work integration policies. The OECD [16] categorized countries into three main groups: (1) Social Democratic, (2) Liberal and (3) Corporatist.

Social Democratic countries were characterized as being relatively the most generous and having the most extensive work integration policies. These countries include Finland, Denmark, the Netherlands, and Norway. In contrast, the Liberal countries were characterized as being relatively the least generous and with the least extensive work integration policies. Countries in this category are the United Kingdom, the United States and Canada. Corporatist countries are characterized being relatively moderate – they were not as generous as the Social Democratic countries but not as conservative as the Liberal countries with respect to benefits and work integration policies. These countries include Austria, Belgium, and France.

Because they are emerging welfare states, the Latin American countries generally are treated as a unique cluster [21,22].

### Gap in the literature

There is a scarcity of literature in which the incidence rates of mental illness-related sickness absence among different countries have been considered together. Part of this gap in the literature may reflect the challenge introduced by the heterogeneity with which countries approach sickness absence. The OECD classification system offers a way to describe systemic similarities and differences among countries. In turn, this information can be used as a first step toward studying effective systemic practices. The purpose of this systematic literature review is to take this first step. We address the question: Are there similarities and differences in the incidence rates of mental disorder-related sickness absence among and within OECD categories? In this paper, we seek to identify differences and similarities rather than to explain them. With this review, we lay the groundwork for and point to areas for future research. In doing so, we also raise questions regarding reasons for the differences and similarities.

## Methods

For the purposes of this systematic review, five electronic databases were searched. They included: (1) *Medline Current* (an index of journal articles in biomedical research and clinical sciences), (2) *Medline In-process* (an index of journal articles in biomedical research and clinical sciences that are awaiting indexing into *Medline Current*), (3) *PsycINFO* (an index of journal articles, books, chapters, and dissertations in psychology, social sciences, behavioral sciences, and health sciences), (4) *Econlit* (an index of journal articles, books, working papers and dissertations in Economics) and (5) *Web of Science* (an index of journal articles, editorially selected books and conference proceedings in life sciences and biomedical research). A search strategy was developed and executed for each database with the help of a professional health science librarian (SB). *Medline Current*, *Medline In-process* and *PsycINFO* were searched using the OVID platform. *Econlit* and *Web of Science* were searched using the ProQuest and Thomson Reuters search interface, respectively. The search was completed between February 2013 and March 2013 and was limited to English language journals published between 2002 and 2013. The complete search strategy used for each database can be found in Appendix 1.

### Eligibility criteria

The systematic literature search focused on working adults between 18–65 years old who had not retired and who had mental and/or substance abuse disorders. Intervention studies were excluded. The search focused on medically certified sickness absences that included sick leave, short-term disability leave, long-term disability leave or sickness absence. For the purposes of this review, sickness absence was defined as a work absence requiring a medical certification. These income replacement or disability benefits (i.e., short-term or long-term work disability) could be either publicly or privately sponsored. In terms of cause of disability, we focused our search on “no cause” disability leaves. That is, the worker did not need to prove that the disability was caused by work.

All search results were screened by title, followed by abstract and full-text review for relevant articles. The reference lists of the articles that made it to the full-text review stage were also hand-searched. The screening process was completed independently by two reviewers, CSD and DL, using the following eligibility criteria:

1. The study reported on medically certified sickness absences due to mental illness and/or addiction problems.

2. The study reported the incidence of medically certified sickness absences due to mental illness and/or addiction problems.

3. The study analyzed data collected in the year 2000 or later.

4. The study sample was not from a select population (i.e. clinical trial, clinical populations).

The year 2002 was used as the starting point for inclusion because the 1990s were a period of global change in employment policies [23]. Thus, we focused on the last decade because during this time, there were relatively fewer policy changes related to workers. Because pre-2000 data were collected under systems that existed before the policy changes of the 1990s, studies that used pre-2000 data were also excluded.

Discussions were held in instances where there were disagreements until consensus was reached. The inter-rater reliability which corrected for chance agreement was calculated for CSD and DL to be 0.93. Review articles and commentaries were excluded when possible during the screening process. Consensus regarding the inclusion of the final articles was reached among CSD, DL and HH.

### Quality assessment

Articles that passed the three-stage screening process were assessed for quality using the following criteria:

1. The study population is well described.

2. The data source is well described.

3. The study sample is representative of the target population.

4. Mental disorders are included and reported.

5. The system of diagnosis/classification is described.

6. The criteria for sickness absence is reported (i.e., pre-sickness absence days to qualify for sickness absence).

7. The denominator is clearly reported.

8. The numerator is clearly reported.

9. Uncertainty of estimates is reported.

10.  The stated research objective is met.

One point was awarded for each met criterion for a maximum score of 10. Scores between 1 and 4 were regarded as ‘fair/weak’ quality and scores between 5 and 8 were ‘good’. Scores of 9 and 10 were regarded as ‘excellent’ quality.

## Results

### Description of inclusion and exclusion

**Figure 1:**
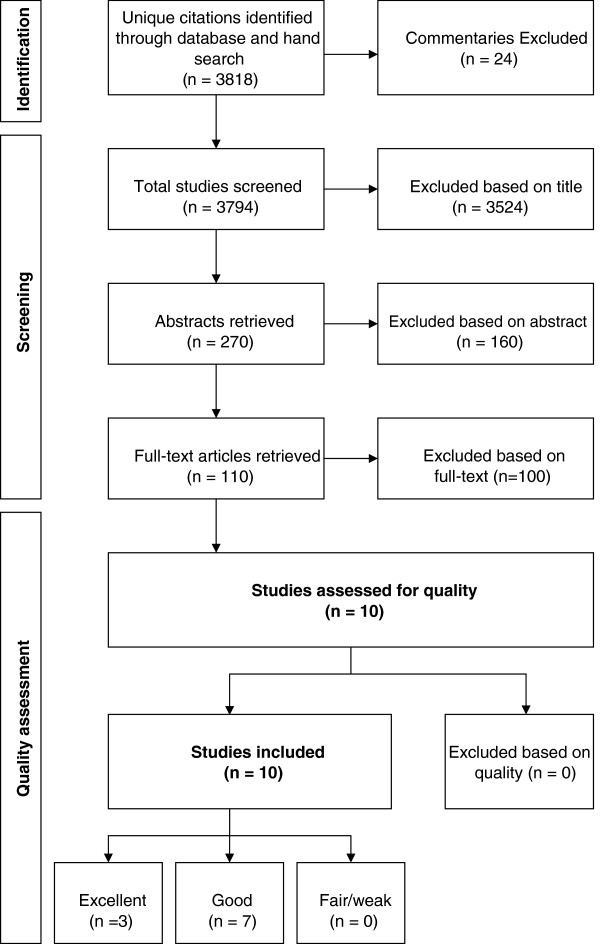
Flowchart of literature search results and inclusions/exclusions.

The electronic literature search resulted in the identification of 3,818 unique citations (Figure 1). From these, 24 entries that were commentaries were excluded. Based on the title review, 3,524 citations were excluded. Based on the abstract review, another 160 citations were excluded; this left 110 articles for full-text review. After the full-text review, 10 articles remained. Reasons for article exclusion included: (1) did not have information about medically certified sickness absence related to mental disorders (n = 33), (2) were based on select populations (n = 13), (3) used pre-2000 data (n = 3), (4) did not report incidence rates from medically certified sickness absence related to mental disorders (n = 49) and (5) the study population did not consist of adults eligible for sickness absence (e.g., the study population included people who were not employed) (n = 2).

The 10 included studies were conducted in countries that clustered into three country types: (1) Social Democratic (n = 6), (2) Liberal (n = 1) and (3) Latin America (n = 3). The Social Democratic category included studies from Norway, Finland and the Netherlands. The study in the Liberal category was from Canada. Finally, all the Latin American studies were from Brazil.

### Quality assessment

The quality assessment rated three of the 10 studies as *excellent* and the remaining seven as *good* (Additional file 1: Table S1, Additional file 2). The identified limitations of these studies include: the study sample was not representative of the target population (8 studies), uncertainty of the incidence rate estimate was not reported (4 studies) and the stated research objective was not met (3 studies).

### Overview of the studies

Table 1 contains the descriptions of the included studies. All of the included studies used administrative data from either an insurer or healthcare group practice. As a result, all of the studies represented identifiable complete populations of people at risk of having a sickness absence and data were either from their sickness absence insurer, workplace or healthcare provider.

**Table 1:** Description of individual studies

**Author(s)**	**Country**	**Study population**	**Data source**	**Year(s) of data**	**Diagnostic classification system used**	**Absence days to qualify for sickness absence benefit**
Social democratic						
Virtanen et al. [24]	FI	Participants from the Finnish Public Sector Study covering employees in 10 towns and 21 public hospitals in Finland; who were not on long-term sick leave or disability pension at the time of the survey; and who were employed for at least 6 months during the study between 1997-2005	Administrative data from the National Health Insurance, employer records and national health register records and the Finnish Public Sector Study	1997-2005	International Classification of Diseases,10^th^ edition (ICD-10)	Long-term sickness absence = sickness absence of ≥ 90 days
Roelen et al. [28]	NL	Employees of firms who were clients of an occupational health services provider from 2001-2007	Administrative sickness absence data from ArboNed	2001-2007	ICD-10	Sickness absence: absence of ≥ 28 sick days requiring a medical certificate from an occupational physician
Koopmans et al. [26]	NL	Dutch Post and Telecommunication employees from 2001-2007	Administrative sickness absence data from ArboNed	2001-2007	ICD-10	Sick leaves of > 3 weeks require a medical certificate from an occupational physician
Roelen et al. [27]	NL	Dutch Post and Telecommunication employees from 2001-2007	Administrative sickness absence data from ArboNed	2001-2007	ICD-10	Sick leaves of > 3 weeks require a medical certificate from an occupational physician
Roelen et al. [29]	NL	Employees covered in a sickness absence benefit program from 2001-2010	Administrative sickness absence data	2001-2010	ICD-10	Sickness absence = absence of > 3 weeks requiring a medical certificate from an occupational physician
Hensing et al. [25]	NO	People who were 16–66 years in 1994, 1996, 1998 and 2000 who were compulsory members of the Sickness Benefit Scheme	Administrative data from the Norwegian National Sickness Administration	1994, 1996, 1998, 2000	International Classification of Primary Care (ICPC)	Medical certification is required for sick leave > 4 days
Liberal						
Dewa et al. [11]	CA (Ontario)	Employees from a large resource sector company from 2003-2006	Administrative sickness absence data	2003-2006	ICD-10	Sickness absence = sickness absence of > 5 continuous work days requiring a medical certificate
Latin America						
Barbosa-Branco et al. [32]	BR	All employees registered in private sector jobs in 2008	Administrative data from health service provider	2008	ICD-10	Sickness absence = ≥ 15 consecutive days absent requiring a medical certificate
Reis et al. [30]	BR	Workers from a university hospital who were employed from 2000-2007	Administrative data from health service provider	2000-2007	ICD-10	Not described
Barbosa-Branco et al. [31]	BR	All employees registered in private sector jobs in 2008	National Benefits System and National Social Information Database	2008	ICD-10	Sickness absence = ≥ 15 consecutive days absent requiring a medical certificate

With the exception of one of the studies, which used the International Classification of Primary Care (ICPC), the studies used the International Classification of Diseases 10^th^ edition (ICD-10). However, Virtanen et al.’s [24] study also reported the ICD-10 equivalents to the ICPC.

There was variability among the included studies with respect to the primary diagnoses of the sickness absence cases that were included in the analyses. However, there were also similarities; all studies included absences related to depressive, anxiety and stress-related disorders. Thus, there appeared to be consistency among the studies with regard to a core set of mental disorders.

There was variability in the number of absence days needed to qualify for sickness absence benefits. The number of qualifying days used for the studies ranged from 3 days to 90 days. It should be noted, that while the days required to qualify for benefits in Finland is 9 days, due to limited availability of the data, the Finnish study [24] examined the incidence of sickness absences that were >90 days.

#### Numerators: measures of sickness absence rates

In general, the studies used two types of incidence measures. The first type of measure reported the incidence of workers with sickness absence. That is, either only the first episode or the worker who had an episode was counted.

In contrast, the second type of measure reported the incidence of sickness absences. It counted the number of episodes occurring in a defined time period. Thus, if a person had more than one sickness absence during the time period of interest, s/he was counted as many times as there was a discrete sickness absence.

#### Cohorts: measures of sickness absence rates

Among the included studies, two types of cohorts were used. One was a 1-year cohort. It included people who were at risk of a sickness absence during a 12-month period. The other was a dynamic cohort for which multiple years of data were used such that the denominator was calculated with the number of workers at risk of sickness absence in terms of either worker-years or worker-months.

### Reported incidence rates

#### Social democratic countries

Using year 2000 administrative data from the national sickness administration, Hensing et al. [25] observed that the age-adjusted cumulative incidence of men with sickness absence ranged from 0.9/1,000 workers for psychosis-related absences to 13/1,000 for depression-related absences (Table 2). In contrast, for women, the cumulative incidence rates ranged from 1/1,000 for psychosis-related absences to 30/1,000 for depression-related absences.

**Table 2:** Results of individual studies

**Author(s)**	**Country**	**Mental disorders**	**Measure**	**Denominator**	**Numerator**	**Reported incidence**
Social democratic						
Virtanen et al. [24]	FI	Mental and behavioral disorders including: depressive disorders, mania and bipolar affective disorder, anxiety disorders (phobias, panic disorder, obsessive compulsive disorder and generalized anxiety disorder), reaction to severe stress and adjustment disorders, personality disorder, schizophrenia, schizotypal and delusional disorders and mental and behavioral disorders due to psychoactive substance use (ICD-10 Chapter F)	Study participants followed for an average of 6.3 years	n = 141,917	Depressive disorder = 2,679	Cumulative incidence of disability benefit receipt:
						Depressive disorders = 1.9%
			Cumulative incidence			
					Mania and bipolar affective disorder =150	Mania and bipolar affective disorder = 0.1%
					Anxiety disorder = 314	Anxiety disorder = 0.2%
					Reaction to severe stress and adjustment disorders = 275	Reaction to severe stress and adjustment disorders = 0.2%
					Adult personality and behaviour disorders = 54	Adult personality and behaviour disorders = 0.04%
					Schizophrenia and schizotypal and delusional disorder = 283	Schizophrenia and schizotypal and delusional disorder = 0.2%
					Mental and behavioural disorders owing to psychoactive substance use = 62	Mental and behavioural disorders owing to psychoactive substance use = 0.04%
Roelen et al. [28]	NL	Common mental disorders (CMD) included distress (ICD-10 R45), other stress-related disorders (ICD-10 F43), depressive disorders (ICD-10 F32) and anxiety disorders (ICD-10 F40 and F41)		Total Employees:	Number of episodes:	12-month incidence of sickness absence for CMD by year/100 employees (95% CI):
			Dynamic cohort study 12-month incidence of total certified sickness absence = number of medically certified sickness absence episodes/number of employees covered			
				2001 = 956,623	2001 = 21,140	2001 = 2.2 (2.2, 2.2)
				2002 = 962,235	2002 = 22,803	2002 = 2.4 (2.3, 2.4)
				2003 = 937,030	2003 = 24,917	2003 = 2.7 (2.6, 2.7)
				2004 = 1,037,149	2004 = 27,533	2004 = 2.7 (2.6, 2.7)
				2005 = 961,890	2005 = 22,682	2005 = 2.4 (2.3, 2.4)
				2006 = 970,390	2006 = 20,013	2006 = 2.1 (2, 2.1)
				2007 = 921,741	2007 = 18,513	2007 = 2 (2, 2)
Koopmans et al. [26]	NL	Common mental disorders (CMD) from medical certification: stress-related (distress and adjustment disorders) (ICD-10 R45, F43) and psychiatric (mild to moderate depressive and anxiety disorders) (ICD10 F32.0, F32.1, F40.0, F40.1, F40.2, F41.0, F41.1, F41.2, F41.3)	Dynamic cohort study	Number of employees = 137,172		From 2001–2007, CMD densities/1,000 worker-years (95% CI):
			Index episode = one episode during research period	Worker-years = 363,461		
					Men:	Men:
			Incidence density of index episodes = # of employees with a first episode of sickness absence due to CMDs between 2001 and 2007/worker-years of the total population at risk		Stress = 4,704	Stress = 19.7 (19.1, 20.2)
					Psychiatric = 723 (2.8, 3.2)	Psychiatric = 3.0 (2.8, 3.2)
					Total CMD = 34,603	Total CMD = 21.8 (21.2, 22.4)
					Women:	Women:
					Stress = 3,298	Stress = 27.8 (26.8, 28.7)
					Psychiatric = 612	Psychiatric = 5.2 (4.7, 5.6)
					Total CMD = 18,026	Total CMD = 31.5 (30.5, 32.5)
Roelen et al. [27]	NL	Mental and behavioral disorders from medical certification (ICD-10 F00-F99)	Dynamic cohort	Number of employees = 137,172	Mental and behavioural disorders = 7,197	From 2001–2007, incidence density/1,000 worker-years
						Mental and behavioural disorders (95% CI):
			Incidence density = incident episodes of sickness absence/worker-years at risk	Worker-years = 363,461		
						Incidence density = 27.7 (27.0, 28.4)
Roelen et al. [29]	NL	Mental and behavioral disorders from medical certification: emotional disturbance (ICD-10 R45), depressive disorders (ICD-10 F32), anxiety disorders (ICD-10 F40-41) and stress-related disorders (ICD-10 F43)	Incidence/year	2001 = 956,623	Not described	Incidence of sickness absence by year/1,000 employees (95% CI):
						2001 = 21.1 (20.8, 21.4)
				2002 = 962,235		2002 = 22.5 (22.3, 22.8)
				2003 = 937,030		2003 = 25.3 (25.0, 25.6)
				2004 = 1,037,149		2004 = 25.5 (25.2, 25.8)
				2005 = 961,890		2005 = 22.9 (22.6, 23.2)
				2006 = 970,390		2006 = 20.0 (19.7, 20.3)
				2007 = 913,266		
						2007 = 20.1 (19.8, 20.4)
				2008 = 924,300		
						2008 = 19.4 (19.1, 19.7)
				2009 = 1,033,072		
						2009 = 16.9 (16.6, 17.2)
				2010 = 1,006,861		
						2010 = 17.7 (17.4, 18.0)
Hensing et al. [25]	NO	Included: Psychoses (ICD-10 F20-31, F35-39), anxiety (ICD-10 F40-F43), neurotic conditions (ICD-10 F44-48, F99), depression (ICD-10 F32-F34), personality disorders (ICD-10 F60-69), alcohol/drug abuse (ICD-10 F10-F19)	Cumulative incidence = # of individuals with ≥ 1 sickness absence episode initiated in each year studied/# of individuals entitled to sickness benefits during that year	Denominator:	Not described	Age-adjusted cumulative incidence of sickness absence in 2000 (95% CI):
				Men: n = 1,219,338		
				Women: n = 1,063,423		Men:
						Psychoses = 0.09% (0.09, 0.09)
						Anxiety disorders = 0.20% (0.19, 0.20)
						Neurotic conditions = 0.54% (0.54, 0.54)
						Depression = 1.31% (1.29, 1.33)
						Personality disorders = 0.01% (0.01, 0.01)
		Excluded: Dementia, organic psychoses, mental retardation and child and adolescent psychiatry				
						Alcohol and drug disorders = 0.09% (0.09, 0.09)
						Women:
						Psychoses = 0.10% (0.10, 0.10)
						Anxiety disorders = 0.35% (0.34, 0.35)
						Neurotic conditions = 1.11% (1.09, 1.13)
						Depression = 3.01% (3.00, 3.04)
						Personality disorders = 0.01% (0.01, 0.02)
						Alcohol and drug disorders = 0.02% (0.02, 0.03)
Liberal						
Dewa et al. [11]	CA (Ontario)	Schizophrenia, mood disorders, stress-related disorders and mental and behavioral disorders due to psychoactive substance use (ICD-10 F00-F99 and Z502, Z503, Z561-566, Z630-Z639, Z729, Z733, Z738, Z864 and Z915)	Incidence = Number of sickness absence episodes/worker-years at risk	n = 12,407 employees	Total = 698	Incidence of disability/100 worker-years (95% CI):
				n = 33,028.79 worker-years	Men = 449	Mental disorders:
					Women = 249	Total = 2.1 (2.0, 2.3)
						Men = 1.7 (1.6, 1.9)
						Women = 3.6 (3.2, 4.1)
Latin America						
Barbosa-Branco et al. [32]	BR	Disorders in the ICD-10 Mental and Behavioral Disorders Chapter 5	Case = a newly granted sickness absence claim	n = 32,590,239	Not described	Age and sex standardized rates of sickness absences for mental and behavioral disorders/10,000 workers = 45.1
			Cases that were within 60 days of each other for the same diagnosis were considered to constitute one case			
			Incidence = number of sickness benefit claims due to mental disorders/average number of workers at risk			
Reis et al. [30]	BR	Mental and behavioral disorders (ICD-10 F00-F99)	Incidence density = number of new sickness absence/total worker-time at risk for the first sickness absence	n = 1,542 workers	n = 324	Mental and behavioral disorders:
						Incidence density/100 worker-months = 0.33
Barbosa-Branco et al. [31]	BR	Disorders in the ICD-10 Mental and Behavioral Disorders Chapter 5	Case = a newly granted sickness absence claim	n = 32,590,239		Prevalence of sickness absence claims/10,000 workers:
			Cases that were within 60 days of each other for the same diagnosis were considered to constitute one case		Any mental disorder = 147,105	Any mental disorder = 45.1
					Depressive episode = 50,289	Depressive episode = 15.4
					Other anxiety disorder = 19,508	Other anxiety disorder = 6.0
			Incidence = number of sickness benefit claims due to mental disorders/average number of workers at risk		Recurrent depressive episode = 14,524	Recurrent depressive episode = 4.5
					Multiple drug use = 11,224	Multiple drug use = 3.4
					Bipolar affective disorders = 9,504	Bipolar affective disorders = 2.9
					Reaction to severe stress = 9,008	Reaction to severe stress = 2.8
					Use of alcohol = 8,545	Use of alcohol = 2.6
					Schizophrenia = 4,616	Schizophrenia = 1.4
					Use of cocaine = 3,468	Use of cocaine = 1.1
					Unspecified nonorganic psychosis = 2,950	Unspecified nonorganic psychosis = 0.91
					Phobic anxiety disorders = 2,023	Phobic anxiety disorders = 0.6
					Unspecified nonorganic psychosis = 1,794	Unspecified nonorganic psychosis = 0.6

Virtanen et al. [24] used linked data from 1997–2005 and reported the cumulative incidence of long-term sickness absence that ranged from 2/1,000 for absences related to schizophrenia and schizotypal and delusional disorders to 19/1,000 for absences related to depression.

Using data from 2001–2007 from one organization with a nation-wide employee base, Koopmans et al. [26] reported an incidence density of 21.8/1,000 worker-years for common mental disorders (CMD) among men and 31.5/1,000 among women. Using a similar dataset, Roelen et al. [27] observed an incidence density of 27.7/1,000 worker-years for mental and behavioral disorders.

Based on data from an occupational health service provider, Roelen and colleagues [28] calculated the 12-month incidence rates for sickness absences related to CMD from 2001–2007. During that time period, it appeared that 12-month incidence rates related to CMD ranged from a high of 27/1,000 employees in 2003 and 2004 to 20/1,000 employees in 2007. In a separate study, Roelen et al. [29] estimated that 12-month incidence rates related to mental and behavioral disorders ranged from 21.1/1,000 employees in 2001 to 17.7/1,000 employees in 2010.

#### Liberal countries

Based on data from one organization with a province-wide employee base, Dewa et al. [11] reported a rate of 21/1,000 worker-years for sickness absences related to mental disorders. When stratified by sex, the incidence rate for men was 17/1,000 worker-years and 36/1,000 worker-years for women.

#### Latin America

All of the Latin American studies in this review were from Brazil. Using 2000–2007 data from one university hospital, Reis and colleagues [30] reported an incidence density of 0.33/100 worker-months or approximately 39.6/1,000 worker-years. Based on 2008 national data of private sector companies, Barbosa-Branco et al. [31,32] observed an incidence rate of 45.1/10,000 or 4.5/1,000 workers. For specific mental disorders, incidence rates varied by primary disorder from 15.4/10,000 workers (1.5/1,000) for sickness absences related to depression and 2.8/10,000 (0.3/1,000) workers for reaction to severe stress.

## Discussion

The quality ratings of included studies ranged from *good* to *excellent*. There was variation in the incidence rates reported by the studies from the Social Democratic, Liberal and Latin American countries in this review. For the studies conducted in the Social Democratic countries, the incidence rates of sickness absences related to mental disorders ranged between 19 and 28/1,000 workers or 1,000 worker-years. In contrast, the incidence rate reported by the study from the Liberal country was 21/1,000 workers. In addition, there was variation in the rates reported by the studies from the Latin America and ranged from 2 - 40/1,000 workers.

The differences reported raise a number of questions. Are they a reflection of the differences in system structures? For instance, among countries in which there is relatively more government involvement (i.e., Social Democratic), the range in the incidence rates is relatively small. The Liberal country group consisted of one country. Yet, the Liberal countries have the least government involvement in benefit and work integration schemes. If that is the case, sickness absence rates are based on definitions of work disability and benefit qualification criteria that depend on private schemes that could be as varied as they are numerous. Such variation in schemes could in turn impact the variation in rates. To further pursue this line of inquiry and to understand variations within country types, it will be useful if a typology similar to the OECD’s [16] were developed especially in countries where there is less government involvement. In addition to describing countries, such a typology could characterize sickness absence benefit schemes.

Another question that arises is why there was such variation among the Latin America studies given they were all from Brazil? Was it because there are significant differences in the mental health among workers? Or are there significant differences in the benefit schemes (i.e., qualification criteria)? Here, a typology characterizing the benefit generosity (i.e., the population covered, disability benefit eligibility and criteria) and work integration schemes (i.e., employer obligations for sickness absence, employment rehabilitation programs and work incentives) of individual plans could assist in answering these questions.

The results also indicate that among the studies that report incidence rates by sex, there is a trend toward a higher incidence rate among women than men. This corroborates findings from Hensing and Wahlstrom’s [33] systematic review of risk factors associated with sickness absence. They found evidence suggesting that women have a higher risk of having an absence related to mental disorders.

### Strengths and limitations related to interpreting the literature

There were a number of strengths of the current body of literature reviewed. First, all of the studies represented identifiable complete populations of people at risk of having a sickness absence. At the same time, it is important to note that there was variation in the breadth of the populations covered from entire countries to single organizations. Thus, it will be important for future work to examine whether the rates reported hold for larger populations and for different populations within the same country.

Another strength was that all of the studies used standardized diagnostic classification systems. All included depressive and anxiety disorders as well as stress-related disorders. However, there was variability in the other type of disorders considered. This could have made some rates higher than others. At the same time, it should be noted that the majority of sickness absences related to mental disorders are attributable to depression, anxiety and stress-related disorders [34,35]. This suggests that inclusion of these disorders would capture a large proportion of the sickness absences related to mental disorders.

A limitation of the studies was the variation in the years they captured. Although all studies used post-2000 data, there could have been changes within systems that could have affected incidence rates. For example, in the Netherlands, extensive legislative changes occurred between 2000 and 2013 which affected rates [23,36]. In fact, the changes are reflected in the rates reported by Roelen et al. [29]. Similarly, changes could have been implemented in other countries such that rates could vary depending on year.

Another limitation was variability in the absence days cut-offs used. A low number of qualifying days could have made the rates higher compared to benefit schemes with a greater number of qualifying days. At the same time, all schemes required medical certification and an assessment of work ability. To the extent that symptoms manifest over several weeks, it may be that workers seek a sickness absence at similar phases of their mental disorder. If the acute phase at which they apply for a sickness absence leave is similar, the actual variation may be minimized. On the other hand, if there is variation in when workers seek medical certificates, incidence rates may be higher in studies where medical certification takes place in an earlier phase of sickness absence. However, the results of Roelen et al.’s [29] study did not seem to support the latter hypothesis. But, this suggests another area for future inquiry – when do workers apply for sickness absence?

### Strengths and limitations of the search strategy

While five databases were searched, it is possible that an article could have been missed if it did not appear in any of the databases. However, that possibility is small given the broad scope of each of the databases. Another limitation is that the search was limited to English-language journals. Thus, it did not identify research that was not published in English. However, it should be noted that despite the language constraint, the included studies came from Europe, North America and Latin America. This suggests that at least some of the researchers from countries in which English is not a first language are publishing in English-language journals.

#### Future directions

Both the causes of and the effective return to work strategies for sickness absences related to mental disorders are multifactorial and complex [37,38] and extend beyond the scope of this paper. Indeed, the results of this paper raise more questions than they answer. Why are there differences among country types? Do these differences truly exist? Or, are they anomalies of the data used? What role does the sickness absence benefit structure play in the incidence rates? What is the appropriate benchmark for sickness absence related to mental disorders?

Cooperation and data sharing among countries as well as between database holders and researchers could help to increase the understanding regarding the similarities and differences of incidence rates. Access to the data necessary to calculate incidence rates often presents a challenge [39,40]. Rather than relying on primary data collection, these types of studies rely on administrative data. This means that the researchers are often not involved in the dataset design. As a result, the calculation of incidence estimates is often influenced by the data limitations. In the future, it would be useful if data warehouses were created where data necessary for this type of research were accessible. It would also help to advance the field if the database managers and researchers were able to work together to design databases that meet administrative and research needs. This would help to promote understanding of incidence rates for a broader range of workers and increase interpretability of the international literature.

## Conclusions

The results of this systematic review suggest that this is an emerging area of inquiry that needs to continue to grow. This review identified only 10 studies that were published in the last 10 years; four of them came from a single country. As this literature continues to expand and if countries are to learn from one another, cross jurisdictional collaboration should be pursued and supported. Perhaps, it could begin among countries categorized in the same OECD category. In addition, as benefit schemes respond to economic circumstances, it will be important that this literature be updated to reflect these changes. Finally, to facilitate a meaningful international dialogue regarding sickness absence, the development of a typology characterizing the sickness absence benefit generosity and work integration policies of sickness absence schemes should be a research priority.

## Appendix 1: Search strategy

### Database: Medline Current

Search Terms: [exp Mental Disorders/OR exp Mentally Ill Persons/OR (mental adj3 disorder$).mp. OR (mental$ adj3 ill$).mp. OR (psychiatric$ adj3 disorder$).mp. OR (psychiatric$ adj3 ill$).mp. OR exp Substance-Related Disorders/OR exp “Diagnosis, Dual (Psychiatry)”/OR (concurrent$ adj3 disorder$).mp. OR (dual$ adj3 diag$).mp. OR (alcohol$ adj3 abus$).mp. OR (alcohol$ adj3 depend$).mp. OR (substance$ adj3 abus$).mp. OR (substance$ adj3 depend$).mp. OR (drug$ adj3 abus$).mp. OR (drug$ adj3 depend$).mp. OR addiction$.mp.] **AND** [exp Absenteeism/OR exp Sick Leave/OR exp Return to Work/OR exp Personnel Turnover/OR Social Welfare/OR Public Assistance/OR exp Insurance Disability/OR exp Insurance Benefits/OR exp Salaries/OR exp Fringe Benefits/OR exp Social Security/OR exp Retirement/OR (sick$ adj3 day$).mp. OR (illness$ adj3 leave$).mp. OR (disabilit$ adj3 leave$).mp. OR (short term disabilit$).mp. OR (long term disabilit$).mp. OR (work$ adj3 absence$).mp. OR (return$ to work$).mp. OR (work$ adj3 turnover$).mp. OR (employ$ adj3 turnover$).mp. OR (disabilit$ benefit$).mp. OR (employ$ benefit$).mp. OR (work$ benefit$).mp. OR (sick$ benefit$).mp. OR (incapacit$ benefit$).mp. OR (social$ welfar$).mp. OR (public$ assistanc$).mp. OR (insurance$ disabilit$).mp. OR (insurance$ benefit$).mp. OR (old$ age$ assistanc$).mp. OR (social$ securit$).mp. OR retire$.mp.] **AND** [sn.fs. OR ep.fs. OR preval$.mp. OR incid$.mp. OR statistic$.mp. OR exp Epidemiologic Methods/].

### Database: Medline In-process

Search Terms: [exp Mental Disorders/OR exp Mentally Ill Persons/OR (mental adj3 disorder$).mp. OR (mental$ adj3 ill$).mp. OR (psychiatric$ adj3 disorder$).mp. OR (psychiatric$ adj3 ill$).mp. OR exp Substance-Related Disorders/OR exp “Diagnosis, Dual (Psychiatry)”/OR (concurrent$ adj3 disorder$).mp. OR (dual$ adj3 diag$).mp. OR (alcohol$ adj3 abus$).mp. OR (alcohol$ adj3 depend$).mp. OR (substance$ adj3 abus$).mp. OR (substance$ adj3 depend$).mp. OR (drug$ adj3 abus$).mp. OR (drug$ adj3 depend$).mp. OR addiction$.mp.] **AND** [exp Absenteeism/OR exp Sick Leave/OR exp Return to Work/OR exp Personnel Turnover/OR Social Welfare/OR Public Assistance/OR exp Insurance Disability/OR exp Insurance Benefits/OR exp Salaries/OR exp Fringe Benefits/OR exp Social Security/OR exp Retirement/OR (sick$ adj3 day$).mp. OR (illness$ adj3 leave$).mp. OR (disabilit$ adj3 leave$).mp. OR (short term disabilit$).mp. OR (long term disabilit$).mp. OR (work$ adj3 absence$).mp. OR (return$ to work$).mp. OR (work$ adj3 turnover$).mp. OR (employ$ adj3 turnover$).mp. OR (disabilit$ benefit$).mp. OR (employ$ benefit$).mp. OR (work$ benefit$).mp. OR (sick$ benefit$).mp. OR (incapacit$ benefit$).mp. OR (social$ welfar$).mp. OR (public$ assistanc$).mp. OR (insurance$ disabilit$).mp. OR (insurance$ benefit$).mp. OR (old$ age$ assistanc$).mp. OR (social$ securit$).mp. OR retire$.mp.] **AND** [sn.fs. OR ep.fs. OR preval$.mp. OR incid$.mp. OR statistic$.mp. OR exp Epidemiologic Methods/].

### Database: *PsycINFO*

Search Terms: [exp Mental Disorders/OR exp Psychiatric patients/OR (mental adj3 disorder$).mp. OR (mental$ adj3 ill$).mp. OR (psychiatric$ adj3 disorder$).mp. OR (psychiatric$ adj3 ill$).mp. OR exp Drug Abuse/OR exp Drug Addiction/OR exp Drug Dependency/OR exp Alcohol Abuse/OR exp Addiction/ OR exp Dual Diagnosis/OR (concurrent$ adj3 disorder$).mp. OR (dual$ adj3 diag$).mp. OR (alcohol$ adj3 abus$).mp. OR (alcohol$ adj3 depend$).mp. OR 321$.cc.[psychological disorders class code] OR 3233.cc.[Substance abuse & addic class code] OR (substance$ adj3 depend$).mp. OR (drug$ adj3 abus$).mp. OR (drug$ adj3 depend$).mp. OR addiction$.mp.] **AND** [exp Employee Absenteeism/OR (absenteeism$).mp. OR exp Employee Leave Benefits/OR exp Reemployment/OR exp Employee Turnover/OR (social welfar$).mp. OR exp Insurance/OR exp Salaries/OR exp employee benefits/OR exp Social Security/OR exp Retirement/OR (sick$ adj3 day$).mp. OR (illness$ adj3 leave$).mp. OR (disabilit$ adj3 leave$).mp. OR (short term disabilit$).mp. OR (long term disabilit$).mp. OR (work$ adj3 absence$).mp. OR (return$ to work$).mp. OR (work$ adj3 turnover$).mp. OR (employ$ adj3 turnover$).mp. OR (disabilit$ benefit$).mp. OR (employ$ benefit$).mp. OR (work$ benefit$).mp. OR (sick$ benefit$).mp. OR (incapacit$ benefit$).mp. OR (social$ welfar$).mp. OR (public$ assistanc$).mp. OR (insurance$ disabilit$).mp. OR (insurance$ benefit$).mp. OR (old$ age$ assistanc$).mp. OR (social$ securit$).mp. OR retire$.mp.] **AND** [preval$.mp. OR incid$.mp. OR statistic$.mp. OR exp Epidemiology/OR ext Data collection/OR epidemiolog$.mp. OR (data collection$).mp. OR survey$.mp. OR questionnair$.mp.].

### Database: *Econlit*

Search Terms: [mental disorder* OR mental disorder* OR mental ill* OR psychiatric* OR concurrent* disorder* OR dual* diag* OR alcohol* OR substance* abus* OR substance* depend* OR drug* abus* OR drug* depend* OR addic*] **AND** [absent* OR sick* OR ill* OR disabilit* leav* OR short term disabilit* OR long term disabilit* OR work* OR absence* OR return* to work* OR work* turnover* OR employ* OR benefit* OR welfar* OR public* assistanc* OR insurance* OR old* age* assistanc* OR social securit* OR retire*].

### Database: Web of Science

Search Terms: [mental disorder* OR mental ill* OR psychiatric* disorder* OR psychiatric* ill* OR concurrent* disorder* OR dual* diag* OR alcohol* abus* OR alcohol* depend* OR substance* abus* OR substance* depend* OR drug* abus* OR drug* depend* OR addiction*] **AND** [absenteeism* OR sick* day* OR illness* leave* OR disabilit* leav* OR short term disabilit* OR long term disabilit* OR work* absence* OR return* to work* OR work* turnover* OR employ* turnover* OR disabilit* benefit* OR employ* benefit* OR work* benefit* OR sick* benefit* OR incapacit* benefit* OR social* welfar* OR public* assistanc* OR insurance* disabilit* OR insurance* benefit* OR old* age* assistanc* OR social securit* OR retire*] **AND** [preval* OR incid* OR statistic* OR epidemiolog* OR data collection* OR survey* OR questionnair*].

## Abbreviations

CMD: Common mental disorders; GP: General practitioner; ICD-10: International Classification of Diseases 10^th^ edition; ICPC: International Classification of Primary Care; OECD: Organization for Economic Co-operation and Development.

## Competing interests

The authors declare that they have no competing interests.

## Authors’ contributions

CSD led the conception, design, data acquisition, analysis and interpretation of the data. DL collaborated on the design, data acquisition and analysis. SB collaborated on the design and data acquisition. HH collaborated on the analysis and interpretation of the data. All authors read and approved the final manuscript.

## Pre-publication history

The pre-publication history for this paper can be accessed here:

http://www.biomedcentral.com/1471-2458/14/205/prepub

## Supplementary Material

Additional file 1: Table S1 Quality Assessment Checklist. The additional file contains the quality checklist criteria used to determine the quality of papers being analyzed for the systematic literature review and the scores for each article. Click here for file

Additional file 2 PRISMA 2009 Checklist. Click here for file
