# Supplementary material for: Incidence rates of sickness absence related to mental disorders: a systematic literature review
Source: BMC Public Health. 2014 Feb 26;14:205. doi: 10.1186/1471-2458-14-205 (PMC3939632; doi:10.1186/1471-2458-14-205)
Supplement: Additional file 1: Table S1 — Quality Assessment Checklist. The additional file contains the quality checklist criteria used to determine the quality of papers being analyzed for the systematic literature review and the scores for each article. [file 1471-2458-14-205-S1.pdf]

Quality Assessment Checklist

Author(s)	Year	1	2	3	4	5	6	7	8	9	10	Total
Barbosa-Branco et al.	2011	1	1	0	1	1	1	1	1	0	1	8
Barbosa-Branco et al.	2012	1	1	0	1	1	1	1	1	0	1	8
Dewa et al.	2010	1	1	0	1	1	1	1	1	1	0	8
Hensing et al.	2006	1	1	1	1	1	1	1	1	1	1	10
Koopmans et al.	2010	1	1	0	1	1	1	1	1	1	0	8
Reis et al.	2011	1	1	0	1	1	0	1	1	0	1	7
Roelen et al.	2009	1	1	0	1	1	1	1	1	1	0	8
Roelen et al.	2010	1	1	0	1	1	1	1	1	1	1	9
Roelen et al.	2012	1	1	1	1	1	1	1	1	1	1	10
Virtanen et al.	2011	1	1	0	1	1	1	1	1	0	1	8
**Total**		10	10	2	10	10	9	10	10	6	7	

Quality Assessment Criteria

1. The study population is well described
2. The data source is well described
3. The study sample is representative of the target population
4. Mental disorders are included and reported
5. The system of diagnosis/classification is described
6. The criteria for sickness absence is reported (i.e., pre-sickness absence days to qualify for sickness absence)
7. The denominator is clearly reported
8. The numerator is clearly reported
9. Uncertainty of estimates is reported
10. The stated research objective is met
